# Isolated Persistent Left Superior Vena Cava, Sick Sinus Syndrome, and Challenging Pacemaker Implantation

**DOI:** 10.1155/2017/9842524

**Published:** 2017-08-24

**Authors:** Hatice S. Kemal, Aziz Gunsel, Levent Cerit, Murat Kocaoglu, Hamza Duygu

**Affiliations:** ^1^Department of Cardiology, Near East University Hospital, Nicosia, Cyprus; ^2^Department of Radiology, Near East University Hospital, Nicosia, Cyprus

## Abstract

Persistent left superior vena cava with absent right superior vena cava is a very rare venous anomaly and is known as isolated PLSVC. It is usually an asymptomatic anomaly and is mostly detected during difficult central venous access or pacemaker implantation, though it could also be associated with an increased incidence of congenital heart disease, arrhythmias, and conduction disturbances. Herein, we describe a dual-chamber pacemaker implantation in a patient with isolated PLSVC and sick sinus syndrome.

## 1. Introduction

Combination of persistent left superior vena cava (PLSVC) and absence of right superior vena cava, known as isolated PLVCS, is a very rare anomaly in adult patients. The incidence of congenital cardiac anomalies and atrial and ventricular arrhythmias is increased as well. Due to difficulties in invasive procedures, both clinicians and sonographers should be alerted to the possible presence of this combined venous anomaly and cardiac arrhythmias. 

## 2. Case Report

**Figure 1:**
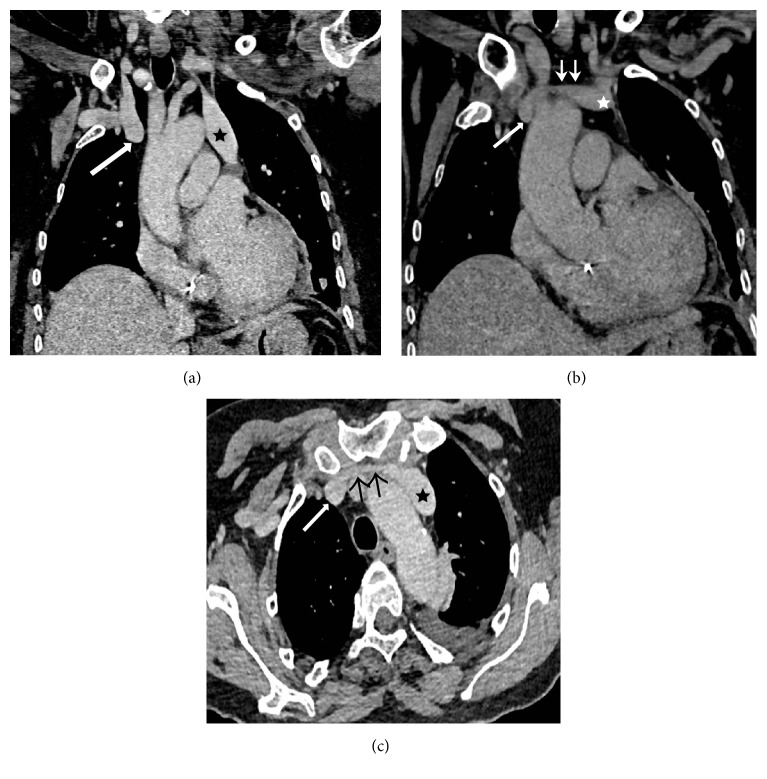
Computer tomography: two consecutive coronal reformatted (a and b) and axial images (c) show stump of the superior vena cava (long arrow), bridging vein (short arrows), and persistent left superior vena cava (star).

A 63-year-old female was admitted to our cardiology department after experiencing a collapse. She was diagnosed with sick sinus syndrome and scheduled for a dual-chamber pacemaker implantation. Echocardiography showed a dilated coronary sinus (CS), suggesting PLSVC, and contrast echocardiography from the left arm vein showed early CS opacification, before the right atrium and right ventricle. A contrast enhanced computer tomography study confirmed persistent left superior vena cava (PLSVC) and absent right superior vena cava. A bridging vein drained the right jugular and right subclavian veins and joined the left brachiocephalic vein in order to form the PLSVC. The PLSVC drained into the right atrium via a dilated CS (Figure 1). 

Leads were placed through the left subclavian vein. Subclavian vein access was performed via direct puncture; the guide-wire was advanced through left subclavian vein to PLSVC and CS to right atrium. Instead of classic lead delivery system, advanced left ventricle lead delivery system for CS cannulation catheter (Medtronic Attain Command + SureValve, Minneapolis, USA) was used to reach the right atrium through CS, for lead placement. Leads were actively fixed in the right atrium (Medtronic CapSureFix MRI active fixation 58 cm lead, Minneapolis, USA) and right ventricle apex with alpha loop configuration (Medtronic CapSureFix MRI active fixation 58 cm lead, Minneapolis, USA). Excellent stimulation and sensing parameters (0.5 V/0.5 V ms for both electrodes, P wave 3 mV, R wave 8 mV) were achieved on both electrodes.

**Figure 2:**
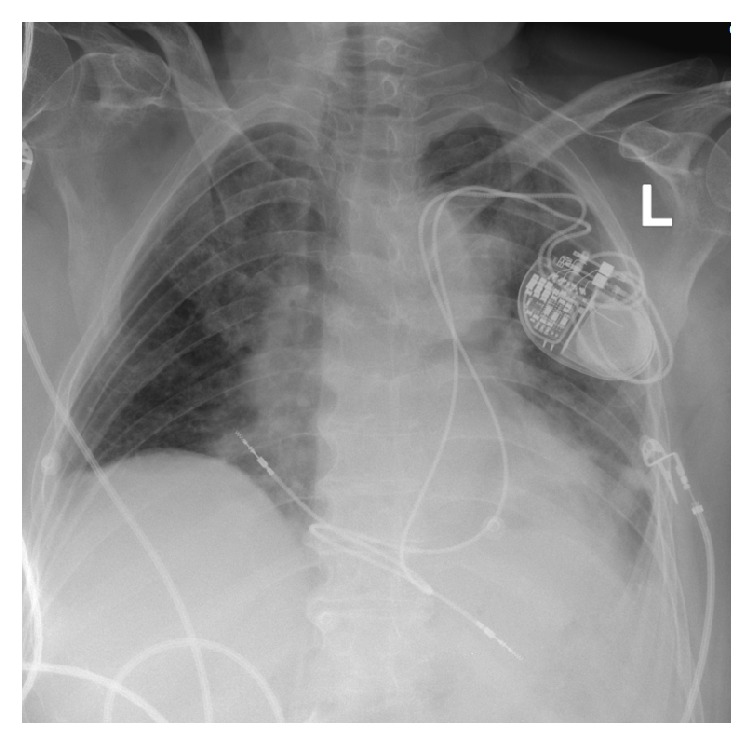
Chest X-ray showing final position of pacemaker and leads.

The patient tolerated the procedure well and there were no complications. A chest X-ray obtained after procedure showed satisfactory lead positioning and no pneumothorax (Figure 2). At follow-up, she was noted to be in good health and her pacemaker was functioning normally.

## 3. Discussion

PLSVC is the most frequent variation of the thoracic venous system, with a prevalence of 0.3–0.5% in general population [1]. PLSVC as a single superior vena cava, in the absence of right superior vena cava, that drains the cephalic portion of the body including upper extremities is much rarer, occurs in 0.09–0.13%, and is known as isolated PLSVC [2].

Presence of a dilated CS on echocardiography should alert the clinician towards the possibility of PLSVC. Computer tomography (MDCT and venography) will confirm the diagnosis [3].

It is also known that patients with PLSVC have higher incidence of arrhythmias and congenital heart defects [4, 5]. Lenox et al. have found sinoatrial node abnormalities in some patients with absent right superior vena cava [4]. The incidence of congenital cardiac anomalies is increased with absence of the right superior vena cava, such as atrial and ventricular septal defect, tetralogy of Fallot, bicuspid aortic valve, aortic coarctation, and cor triatriatum [6].

Right ventricular lead placement via the PLSVC and CS can be technically challenging due to the anomalous anatomy [7]. Serious complications have been reported when a guide-wire or catheter is manipulated via PLSVC, such as angina, arrhythmia, cardiogenic shock, and even cardiac arrest [2]. The use of advanced lead delivery systems and longer leads to reach right atrium and ventricle is needed. In our case, we have used advanced left ventricle lead delivery system for CS cannulation catheter, which enabled us to easily reach the right atrium and provided us more possibilities for manipulation. Also considering preshaped stylet, forming loop within the atrium and active fixation of leads should be kept in mind in order to overcome difficulties. If implantation is impossible despite using multiple techniques, transcatheter pacing system (leadless pacemaker) could be considered.

Although mostly clinically silent, undiagnosed PLSVC can lead to catastrophic consequences during invasive procedures. If PLSVC is suspected, the anatomy of the thoracic venous system must be identified before invasive cardiac procedures.
